# Healthy Immigrant Effect or Under-Detection? Examining Undiagnosed and Unrecognized Late-Life Depression for Racialized Immigrants and Nonimmigrants in Canada

**DOI:** 10.1093/geronb/gbad104

**Published:** 2023-07-27

**Authors:** Shen (Lamson) Lin

**Affiliations:** Department of Social and Behavioural Sciences, City University of Hong Kong, Kowloon, Hong Kong Special Administrative Region, China; Factor-Inwentash Faculty of Social Work, University of Toronto, Toronto, Ontario, Canada; (Social Sciences Section)

**Keywords:** Depression care, Mental health equity, Migration, Minority aging, Race

## Abstract

**Objectives:**

Immigrants to Canada tend to have a lower incidence of diagnosed depression than nonimmigrants. One theory suggests that this “healthy immigrant effect (HIE)” is due to positive selection. Another school of thought argues that the medical underuse of immigrants may be the underlying reason. This unclear “immigrant paradox” is further confounded by the intersecting race–migration nexus.

**Methods:**

This population-based study analyzed data of participants (*n* = 28,951, age ≥45) from the Canadian Community Health Survey (2015–2018). Multivariable logistic regression was employed to examine associations between race–migration nexus and mental health outcomes, including depressive symptoms (Patient Health Questionnaire [PHQ-9] score ≥10).

**Results:**

Compared to Canadian-born (CB) Whites, immigrants, regardless of race, were less likely to receive a mood/anxiety disorder diagnosis (M/A-Dx) by health providers in their lifetime. Racialized immigrants were mentally disadvantaged with increased odds of undiagnosed depression (Adjusted odds ratio [AOR] = 1.76, 99% Confidence interval [CI]:1.30–2.37), whereas White immigrants were mentally healthier with decreased odds of PHQ depression (AOR=0.75, 99%CI: 0.58, 0.96) and poor self-rated mental health (AOR=0.56, 99% CI=0.33, 0.95). Among the subpopulation without a previous M/A-Dx (*N* = 25,203), racialized immigrants had increased odds of PHQ depression (AOR = 1.45, 99% CI: 1.15–1.82) and unrecognized depression (AOR = 1.47, 99% CI: 1.08–2.00) than CB Whites. Other risk factors for undiagnosed depression include the lack of regular care providers, emergency room as the usual source of care, and being home renters.

**Discussion:**

Despite Canadian universal health coverage, the burden of undiagnosed depression disproportionately affects racialized (but not White) immigrants in mid to late life. Contingent on race–migration nexus, the HIE in mental health may be mainly driven by the healthier profile of White immigrants and partly attributable to the under-detection (by health professionals) and under-recognition of mental health conditions among racialized immigrants. A paradigm shift is needed to estimate late-life depression for medically underserved populations.

Late-life mental health conditions are increasingly recognized as public health issues that may lead to a higher risk of suicidal ideation, disability, and shortened life expectancy among older populations (Luijendijk et al., 2008; Pérès et al., 2008). It is estimated that the community prevalence of mental health conditions in old age ranges from 17% to 35% worldwide (Andreas et al., 2017; Leles da Costa Dias et al., 2019). Yet, mental health problems often go undiagnosed and undertreated (Farid et al., 2020; Garrard et al., 1998; Pelletier et al., 2017). A meta-analysis has revealed that there was reasonable evidence of inadequate clinical identification of late-life depression in primary care (Mitchell et al., 2010). Although the diagnosis of a mental health condition is mainly covered by the universal coverage of physician-provided health care in Canada (Steele et al., 2006; Lin, 2023), previous epidemiological studies found that approximately 48% of Canadians reported undiagnosed mood disorders (Pelletier et al., 2017) and around 60% of Canadians with mental health needs did not use any type of mental health care (Vasiliadis et al., 2007).

## The Healthy Immigrant Paradox and Mental Health

Migration is an important social determinant of health (Acevedo-Garcia et al., 2012; How et al., 2021) because it demands a complete realignment of daily life that reshapes opportunities and power differentials to obtain health-enhancing resources, a transition that poses significant challenges to immigrants (Castañeda et al., 2015). Immigrants comprise more than one-fifth of Canada’s population (22%), a percentage that is anticipated to rise to at least 25% by 2031 (Government of Canada, 2020). The rapid growth of immigrants also makes up 65% of populations identified as non-Caucasian in race (hereafter racialized groups), resulting from a larger inflow of humanitarian immigrants such as family reunification arrivals and refugees to Canada from non-European nations (e.g., Philippines, India, China, and Nigeria) over the past half-century (Lin, 2021). With such migration trends continuing, it is estimated that a quarter (26.9%) of the total Canadian population (47.7 million) will be immigrants from Asia or Africa in 2041, up from 13.5% in 2016 (Statistics Canada, 2022). Canada has opened its doors to non-European immigrants since the introduction of the points system in the late 1960s (Triadafilopoulos, 2010). Due to this merit-based points system and the mandatory health screening that favors individuals with established human capital (Laroche, 2000), immigrants to Canada tend to perform better in certain health outcomes than native-born Canadians, particularly for mortality advantage among working-age individuals (Omariba, 2015; Aldridge et al., 2018). This phenomenon has been theorized as the “healthy immigrant effect (HIE),” but such HIE varies for morbidity and across age-related life stages (Vang et al., 2017; Vang & Ng, 2023 ), with inconsistent patterns found for immigrant children and older adults.

The scholarly inquiry about whether HIE extends to mental health outcomes remains controversial and inconclusive in the existing immigrant literature (Elshahat et al., 2021; Hansson et al., 2012). Relying on physician-diagnosed measures, some research suggests that immigrants and racial minority groups have a lower lifetime prevalence of anxiety disorders (Aglipay et al., 2013; Davison, Lin et al., 2020) and mood disorders (Chiu et al., 2018; Nwoke et al., 2020) than their Canadian-born (CB) counterparts. This appears to substantiate the phenomenon of HIE (Whitley et al., 2017). On the contrary, other investigations, based on standardized psychological instruments, refuted the HIE theory and found that immigrants were at elevated risk of poor mental health (Davison et al., 2019; Davison, Lung et al., 2020)—a pattern that aligns more with their experiences of resettlement stressors during the postmigration period (Kirmayer et al., 2011). This unclear “immigrant paradox” is further confounded by the intersecting race–migration nexus (Lee, 2019), as racial inequities in mental illness and mental health care are well documented (Chiu et al., 2018; Sentell et al., 2007).

Another school of thought argues that the medical underuse of immigrants, resulting from barriers to accessing health services (Lin, 2021), may be the underlying reason for this “foreign-born health advantage.” As health outcomes and health care access are inextricably intertwined (Lin, 2022a, b), the mixed findings on mental health disparities are believed to illuminate, among other factors, underlying health care inequities that lead to the underestimation of mental health conditions among migrant communities (Lau et al., 2013). Mounting evidence from scoping and systematic reviews suggest that compared to the host populations, foreign-born residents seem to underutilize mental health services due to limited health literacy (Thomson et al., 2015), linguistic obstacles (Wang et al., 2019), cultural differences (Ahmed et al., 2016), and discrimination in the host country (Edge & Newbold, 2013). In other words, if immigrants are underserved by the receiving country’s health system and hence are less likely to consult with health professionals who would diagnose existing medical conditions, the estimated effect of immigration status on physician-diagnosed mental health conditions at the population level will be biased towards the null (McDonald & Kennedy, 2004). As such, it is a legitimate concern that inequities in health care access would pose a threat to the detection rates of physician-diagnosed mental illnesses for immigrants.

Following this line of inquiry, studies in the United States suggest that physician-diagnosed mental health conditions (e.g., recipients of a mood disorder diagnosis) should serve as proxies for markers of depression care and treatment rather than the health status of patients per se (Akincigil et al., 2012; Vyas et al., 2020). This operationalization is in line with Andersen’s behavioral model of health service utilization (Andersen, 1995; Andersen & Aday, 1978), which specifically conceptualizes “realized access” as the actual use of services. In addition, psychological symptom screeners (i.e., symptom-rating scales) could be employed in contrast to previous clinical diagnoses so that unmet mental health needs for vulnerable populations could be identified (Fan et al., 2009). A meta-analysis revealed that “comparisons of self-reported and diagnosed estimates” should be the focus of future research (Edwards et al., 2019). A growing body of international literature has extended this approach to investigate the prevalence of “undiagnosed mental health conditions,” whereby individuals have current symptoms but without a history of relevant psychiatric diagnoses in the primary care clinical setting (Downey et al., 2012; Vermani et al., 2011), as well as in the community setting (Li et al., 2009; Pelletier et al., 2017; Williams et al., 2017). The nuanced conceptualization of “under-detection,” arising from attitudinal or structural barriers to care, captures the interwoven nature of health outcomes and realized access to health services.

## Research Gaps and Hypotheses

Although mental health inequalities are well documented in relation to race/ethnicity (Chiu et al., 2018) and nativity (Davison et al., 2019; Lin et al., 2020), prior research has typically examined these health differences separately. Although a recent study found that undiagnosed depression was higher among female immigrants (Farid et al., 2020), the immigrant health literature tends to homogenize the experience of racialized and White immigrants as a monolithic category (Brown, 2018; Lin & Fang, 2023). The intersectionality lens of racialization, migration, and old age has been largely overlooked in mental health epidemiology (Gkiouleka et al., 2018; Lee, 2019; Lin, 2023). Moreover, racialized immigrants were often compared with their native-born peers of the same ethnic origin (e.g., Alvarez et al., 2019), rather than using domestically born Whites as the reference category. Ignoring the racial heterogeneity among immigrant populations and the failure of referencing with the dominant privileged group are both serious shortcomings (Lin, 2022b; Lin 2023), because racism, nativism, language barriers, and cross-cultural differences may cumulatively influence the health and well-being of immigrants who are aging in a foreign land (Ferrer et al., 2017).

Therefore, a disaggregated investigation of how intersecting stratification axes of race/ethnicity and nativity combine to shape mental health disparities in late life is warranted to inform culturally responsive mental health services (Lin, 2022b). The current study aims to examine racial-nativity disparities in mental health epidemiology to capture both the issue of health care access and the actual occurrence of depression symptoms. Recognizing that minority communities with adverse social determinants may be more susceptible to stressful life events as well as barriers to accessing health care (Lin, 2021), this study investigates the following three research questions (RQ): compared to CB Whites, (RQ1) racialized immigrants would be more likely to screen positive for depressive symptoms and have poor self-rated mental health; (RQ2) whether they would be less likely to receive a mental disorder diagnosis by a health professional; and (RQ3) whether they would be more likely to have undiagnosed depression as well as unrecognized depression in later life.

## Method

### Data Source

The current study was based on the public-use microdata files (PUMF) from the Canadian Community Health Survey—Annual Components (CCHS). The study population was derived from a combined sample of CCHS across four annual survey circles (2015–2018) via the pooled approach that has been widely applied in previous epidemiological studies (Chiu et al., 2018, 2020). The resulting data set could be treated as if it is a random sample from an average population observed over all survey cycles (Quach et al., 2012) because various CCHS survey cycles were independent (Thomas & Wannell, 2009). The CCHS is a cross-sectional survey conducted by Statistics Canada annually that collects information related to health status and health care utilization among the Canadian populations aged 12 and above across all provinces and territories. A detailed explanation of the complete CCHS survey methodology can be found elsewhere (Statistics Canada, 2018). In the present study, only respondents who completed the screening of depression symptoms module were included (*n* = 55,719). As many racialized immigrants coming from the Global South hold diverse ethnocultural perceptions of old age, the sample was further restricted to participants aged 45 and above (*n* = 32,975). Those with missing data on demographics socioeconomic characteristics, and health characteristics, were excluded from the analyses. This yielded a final analytic sample of 28,951.

### Mental Health Outcomes

*Self-assessed depressive symptoms* were measured by the Patient Health Questionnaire (PHQ-9), a nine-item standardized instrument for major depressive disorders that evaluates the frequency of symptoms in the past 2 weeks, such as anhedonia, poor appetite, trouble concentrating on things, self-injurious ideation, feelings of hopelessness, and restless sleep (Spitzer et al., 1999). These nine criteria were taken directly from the *Diagnostic and Statistical Manual of Mental Disorders*, fourth edition. Psychometric research literature has demonstrated that PHQ-9 has diagnostic validity comparable to clinician-administered Primary Care Evaluation of Mental Disorders for depressive disorders (Kroenke & Spitzer, 2002; Spitzer et al., 1999) and represents a reasonable alternative to the Geriatric Depression scale for older populations (Phelan et al., 2010). PHQ-9 has been rigorously validated in the general population (Martin et al., 2006), tested among different ethnic groups and could be administered without adjustment in racially diverse populations (Huang et al., 2006). This nine-item composite measure (range: 0–27) is a 4-point Likert scale with options ranging from “not at all” (=0), “several days” (=1), “More than half the days” (=2) to “nearly every day” (=3). In the present study, a score of ≥10 on PHQ-9 was applied to identify those who are moderately or severely depressed (Fan et al., 2009), and this cutoff score has a sensitivity of 88% and a specificity of 88% for major depression (Kroenke & Spitzer, 2002).

*Clinically diagnosed mental disorders (Clinical detection).* In the chronic condition module, respondents were asked whether they had any psychiatric disorders (yes/no), including a mood disorder (e.g., *depression, bipolar disorder, mania, or dysthymia*) and/or an anxiety disorder (e.g., *a phobia, obsessive-compulsive disorder, or a panic disorder)*, that are “diagnosed by a health professional and that are expected to last or have already lasted 6 months or more” (Pelletier et al., 2017). These self-reported items had shown good validity in population studies (Sanchez-Villegas et al., 2008). In the Canadian context, only licensed mental health professionals including general practitioners (or family physicians), psychiatrists, and psychologists are authorized to provide formal diagnosis of a mental health problem. As “universality” is the founding principle of the Canada Health Act, the health care system provides coverage, on uniform terms and conditions, for all Canadian citizens and landed immigrants. Yet, the accessibility of mental health care is shaped by which services are government-funded, because Canada has a two-tier mental health care system (Lin, 2023): public taxation (i.e., Medicare) mainly covers physician- and psychiatrist-provided services, whereas private professionals such as clinical psychologists and psychotherapists are financed through job-based supplemental health insurance and consumers’ out-of-pocket payments (Bartram & Stewart, 2019).

*Undiagnosed depression* was defined as respondents having current moderate-to-severe depressive symptoms (PHQ-9 score ≥ 10) but not reporting previous mood disorder or anxiety disorder diagnosis by a health provider. A similar operationalization has been used in both clinical (Vermani et al., 2011) and community settings (Williams et al., 2017).

*Self-rated mental health* (SRMH) was assessed by a question asking, “In general, how would you describe your mental health?” It is a five-point Likert scale with options ranging from excellent, very good, good, fair, and poor (McAlpine et al., 2018). SRMH was predictive of psychiatric conditions, self-rated health as well as perceived needs for professional help (Ahmad et al., 2014).In this study, poor SRMH was created as a binary variable, indicating poor compared to good (good, very good, excellent, or fair) SRMH so as to increase the sensitivity in detecting self-recognition of mental discomfort.

*Unrecognized depression* was theorized as respondents having current moderate-to-severe depressive symptoms (PHQ-9 score ≥ 10) but not reporting “poor” in SRMH. This is a novel conceptualization in the current study to understand whether there are variations in the recognition and self-awareness of depressive symptoms as signs of poor mental health among different social groups (Assari, 2018; McAlpine et al., 2018) such as racial/ethnic disparities (Kim et al., 2011).

### Main Independent Variable

#### Race–migration nexus

Based on participants’ self-identified racial/cultural backgrounds and migration status in the CCHS, racial-nativity status was conceptualized as a key structural driver of inequalities in this study that reflects the social stratification process of racialization and migration experiences in shaping power differentials (Lin, 2021), as a result of which institutional racism and nativity-based systemic discrimination may arise (Gkiouleka et al., 2018). This intercategorical construct classified respondents into four social positionings: (1) CB Whites (reference category), (2) CB non-Whites, (3) Foreign-born (FB) Whites (i.e., Whites immigrants), and (4) racialized immigrants (i.e., FB non-Whites). This variable was regarded as more than individual attributes but as a product of power structures that “rank people into social hierarchies and (re)distribute social determinants of health” (Gkiouleka et al., 2018). The rationale for choosing CB Whites as the reference category is that the intersecting power axes of race and migration jointly reflect a social location of privilege, shaping the health care experience in Canada—a “White-settler society” tied to its sociocultural history (Lee & Bhuyan, 2013).

### Covariates

To reduce the possibility of spurious associations between racial-nativity status and mental health outcomes, potential covariates were selected based on the widely used Behavioral Model of Health Services Use (Andersen, 1995) and the Socioecological Model for Older Racialized Immigrants (Lin, 2021, 2023; Lin & Fang, 2023). These covariates include *socioeconomic factors* (education, annual household income, and homeownership), *patient-side and provider-side enabling factors* (lack of a regular health care provider, usual source of care, marital status, primary language spoken at home, living patterns, sense of community belonging, and perceived life stress), and *health behaviors* (current smoking status, past-week physical exercise, and drinking habits). Due to the PUMF constraint, all covariates were categorical measures and detailed response options were given in Table 1.

**Table 1.** Sample Characteristics: Weighted Percentages Stratified by Racial-Nativity Status and by Depression (PHQ ≥ 10) in the CCHS 2015–2018 (*N* = 28,951), Persons Aged ≥45

	Full sample		Race–migration nexus					Depression	
	Unweighted all *N*	Weighted all %	CB White	CB non-White	FB White	FB non-White	Chi-square	PHQ-9 ≥ 10	Chi-square
Unweighted size (*N*)	28,951	100.0%	24,316	290	2,821	1,524	Sig.	1,702	Sig.
Key mental health outcomes									
Depression (PHQ-9 ≥ 10)	1,702	5.6%	6.2%	4.0%	3.8%	4.6%	<.001	—	—
Mood/anxiety disorder Dx	3,748	11.8%	13.5%	10.6%	9.1%	6.3%	<.001	25.2%	<.001
Mood disorders Dx	2,668	8.4%	9.7%	8.0%	6.5%	4.4%	<.001	30.2%	<.001
Anxiety disorders Dx	2,139	6.6%	7.7%	5.9%	4.6%	3.5%	<.001	29.9%	<.001
Poor SR mental health	424	1.4%	1.5%	1.1%	0.8%	1.4%	.005	64.8%	<.001
Undiagnosed depression	725	2.6%	2.7%	2.4%	1.7%	3.3%	<.001	—-	—
Unrecognized depression	1,413	4.7%	5.1%	3.7%	3.3%	4.2%	<.001	—	—
Demographics									
Age							<.001		<.001
45–54	7,171	34.9%	33.8%	44.7%	26.1%	46.6%		6.8%	
55–64	8,711	32.1%	33.8%	30.1%	28.1%	28.0%		6.3%	
65–74	8,090	21.8%	21.1%	17.3%	29.3%	19.3%		3.5%	
≥75	4,979	11.1%	11.3%	8.0%	16.5%	6.1%		3.9%	
Sex							.001		<.001
Male	13,033	47.7%	47.0%	48.7%	48.1%	50.4%		4.3%	
Female	15,918	52.3%	53.0%	51.3%	51.9%	49.6%		6.8%	
Socioeconomic status									
Family annual income							<.001		<.001
<$20k	2,776	6.3%	6.3%	5.6%	5.2%	7.1%		17.5%	
$20k to <$40k	6,025	14.8%	14.6%	7.7%	16.4%	15.1%		7.2%	
$40k to <$60k	5,225	15.7%	15.2%	18.6%	18.8%	15.1%		6.1%	
$60k to <$80k	4,114	15.0%	14.4%	15.4%	14.3%	17.8%		5.4%	
≥$80k	10,811	48.3%	49.5%	52.7%	45.4%	44.8%		3.5%	
Education							<.001		<.001
<Secondary school	5,522	14.5%	15.7%	8.0%	14.2%	10.0%		8.4%	
Secondary school	6,711	22.9%	24.7%	12.5%	20.6%	17.8%		6.9%	
Postsecondary	16,718	62.5%	59.6%	79.5%	65.2%	72.2%		4.5%	
Homeownership							<.001		<.001
Home owner	23,202	81.8%	83.1%	87.0%	81.3%	75.9%		4.3%	
Home renter	5,749	18.2%	16.9%	13.0%	18.7%	24.1%		11.3%	
Patient-side and provider-side enabling factors									
Language spoken at home							<.001		<.001
English and/or French	28,153	91.6%	99.8%	98.7%	83.4%	60.8%		5.4%	
Other languages	798	8.4%	0.2%	1.3%	16.6%	39.2%		8.5%	
Relationship							<.001		<.001
Married	17,327	72.1%	71.2%	62.9%	72.1%	76.4%		4.3%	
Widowed	8,550	19.5%	19.5%	17.5%	22.0%	17.3%		8.4%	
Single	3,074	8.5%	9.2%	19.6%	5.8%	6.3%		10.4%	
Lack of a regular care provider							.15		<.001
No (have a provider)	26,608	93.2%	93.1%	91.5%	93.8%	93.5%		5.4%	
Yes (lack a provider)	2,343	6.8%	6.9%	8.5%	6.2%	6.5%		8.5%	
Usual source of care (USOC)							<.001		<.001
General practitioner (GP)	17,398	62.9%	62.3%	66.6%	64.2%	64.1%		5.0%	
Hospital outpatient clinic	1,266	2.8%	3.4%	2.7%	1.5%	1.4%		6.1%	
Community health center	1,461	3.3%	3.8%	3.7%	2.5%	1.4%		4.4%	
Walk-in clinic	3,862	16.8%	15.5%	14.1%	19.0%	20.7%		6.3%	
Emergency room	2,657	6.5%	7.8%	4.5%	3.9%	2.8%		9.1%	
Some other places	358	0.9%	1.1%	2.1%	0.6%	0.6%		5.1%	
Lack USOC	1,949	6.8%	6.0%	6.4%	8.3%	9.0%		6.2%	
Perceived life stress							<.001		<.001
Not stressful	13,134	40.4%	39.7%	47.2%	43.5%	40.1%		1.3%	
Stressful	15,817	59.6%	60.3%	52.8%	56.5%	59.9%		8.5%	
Sense of community belonging							<.001		<.001
Strong	21,928	73.9%	74.6%	67.0%	71.6%	73.2%		3.7%	
Weak	6,723	25.0%	24.4%	31.6%	26.7%	25.6%		11.2%	
Not stated	300	1.1%	1.0%	1.3%	1.7%	1.1%		6.3%	
Health behaviors									
Body mass index							<.001		<.001
Underweight	331	1.0%	0.9%	0.3%	1.1%	1.3%		9.2%	
Normal weight	9,524	35.8%	33.2%	33.0%	36.6%	46.9%		4.5%	
Overweight	10,677	36.9%	36.7%	39.4%	38.3%	36.1%		4.5%	
Obese—Class I, II, III	7,427	23.1%	25.7%	25.5%	20.3%	13.5%		8.7%	
Not stated	992	3.3%	3.5%	1.9%	3.7%	2.2%		7.9%	
Drinking							<.001		<.001
Regular drinker	16,816	61.0%	64.8%	63.4%	67.8%	38.5%		4.6%	
Occasional drinker	5,208	17.1%	16.5%	22.8%	15.3%	20.9%		7.3%	
Not past-year drinking	6,927	21.8%	18.7%	13.8%	16.9%	40.6%		7.2%	
Smoking							<.001		<.001
Daily smoker	4,116	13.5%	15.6%	12.2%	11.3%	6.5%		12.5%	
Occasion smoker	849	3.1%	3.1%	2.9%	3.5%	2.5%		7.2%	
Nonsmoker	23,986	83.4%	81.3%	84.9%	85.3%	91.1%		4.4%	
Past-week sports							<.001		<.001
Yes	13,977	50.4%	51.2%	47.7%	49.8%	47.3%		3.1%	
No	14,974	49.6%	48.8%	52.3%	50.2%	52.7%		8.1%	

*Notes*: CB = Canadian born; CCHS = Canadian Community Health Survey; FB = foreign-born; PHQ-9 = Patient Health Questionnaire for major depression (PHQ-9 score ≥ 10 indicates moderate-to-severe symptoms); SR = self-reported; Dx = diagnosis.

Those who had a regular care provider includes a family doctor (91.2%), a medical specialist such as a cardiologist or pediatrician (0.6%), and a nurse practitioner (1.1%).

### Statistical Analysis

Unweighted statistics were used to describe the overall sample characteristics. Weights were normalized/standardized to produce estimates corrected for the sample size and to apply equal weights for each survey circle in the pooled data. To calculate normalized weights, the survey weight (of each unit used in the analysis) was divided by the (unweighted) average of the survey weights of all the analyzed units. First, cross-tabulation analyses were generated by Chi-square tests (χ^2^) using weighted percentages to compare between-group differences by four racial-nativity groups and by self-rated depression. Second, binary logistic regression was conducted to examine associations between racial-nativity status and five dichotomous mental health outcomes while adjusting for covariates. Because the fully adjusted models were at risk of multiple comparisons, a more stringent criterion was employed to interpret *p* values for all analyses in this study (*p* < .01). Multicollinearity issue was also checked to make sure the variance inflation factor less than 3 (Vatcheva et al., 2016). Lastly, because the clinical detection of mood/anxiety disorder diagnosis (M/A-Dx) was a confounding variable between race–migration nexus and key mental health outcomes, the multivariable-adjusted logistic regression were then repeated (as sensitivity tests) via stratifying by the presence/absence of M/A-Dx to predict PHQ-9 depression and unrecognized depression, respectively (see Supplementary Material 1). Statistical analyses were performed using the SPSS software package, Version 26 (IBM Corp., Armonk, NY, USA). Model performance was assessed by Nagelkerke’s *R*^2^ statistic.

## Results

### Sample Characteristics

Table 1 summarizes the unweighted sample size and weighted proportion (%) of symptom prevalence, diagnosis rates, and social–demographic covariates. The weighted overall sample (*n* = 28,951) mainly consisted of respondents who were in a relationship (72.1%) and speaking English/French at home (91.6%). Canadian-born Whites accounted for the majority (70%) in the sample, with 1.3% CB non-Whites, 13.1% White immigrants, and 15.7% racialized immigrants. The sex distribution was even (men: 47.7%; women: 52.3%). Most variables were significantly linked to race–migration status, except for access to a regular care provider (*p* = .15). Compared to CB Whites, racialized immigrants were more likely to be home renters (24.1% vs 16.9%), younger age (in the age group of 45 to 54 years old: 46.6% vs 33.8%), well-educated (postsecondary education: 72.2% vs 59.6%) and speaking nonofficial languages at home (39.2% vs 0.2%), lacking a usual place for minor health problems (8.9% vs 6%), and visiting walk-in clinics more often (20.7% vs 15.5%); racialized immigrants tended to have physically healthier profiles, as evident by body mass index measure (normal weights: 46.3% vs 33.2%), no past-year drinking (40.6% vs 18.7%), and nonsmoking (91% vs 81.3%).

**Figure 1.**
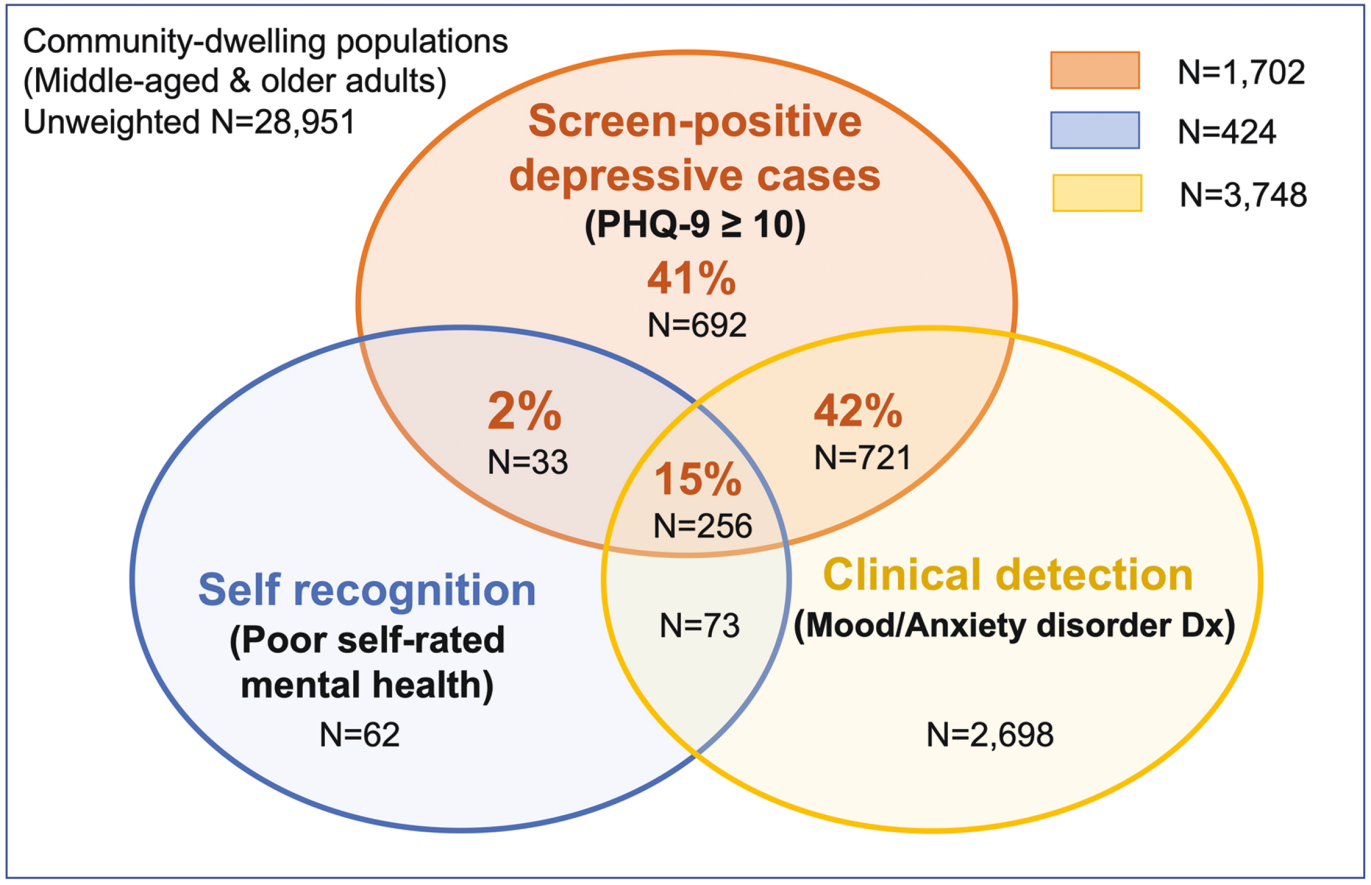
Venn diagram displaying the extent of concordance of PHQ-9 screen-positive depression with clinical diagnosis of mood/anxiety disorder and poor self-rated mental health in the Canadian Community Health Study 2015–2018, persons aged ≥45 (*N* = 28,951). *Notes*: Percentages are based on all PHQ screen-positive depressive cases. The sample size is unweighted. Undiagnosed depression *N* = 725 (43% of positive cases); unrecognized depression *N* = 1,413 (83% of positive cases).

For the key outcome measures, Figure 1 displays the concordance level between screen-positive depression (PHQ-9 score ≥10) with the professional diagnosis of mood/anxiety disorder (clinical detection) and poor SRMH (self-recognition). Of those who screened positive for depressive symptoms, 57% had received a clinical diagnosis of mood/anxiety disorder, 17% rated themselves in poor mental health, and 41% had neither clinical detection nor self-recognition. Among four racial-nativity groups, CB Whites had the highest rate of screen-positive depression (6.2%), followed by racialized immigrants (4.6%), CB non-Whites (4.0%), and White immigrants (3.8%). For clinical detection, CB Whites had the highest diagnosis rates for mood disorders (9.7%) and anxiety disorders (7.7%), whereas racialized immigrants had the lowest diagnosis rates for mood disorders (4.4%) and anxiety disorders (3.5%). Racialized immigrants had the highest prevalence of undiagnosed depression (3.3%), followed by CB Whites (2.7%), CB non-Whites (2.4%), and White immigrants (1.7%).

#### RQ1: Depressive symptoms (PHQ-9) and poor SRMH

**Figure 2.**
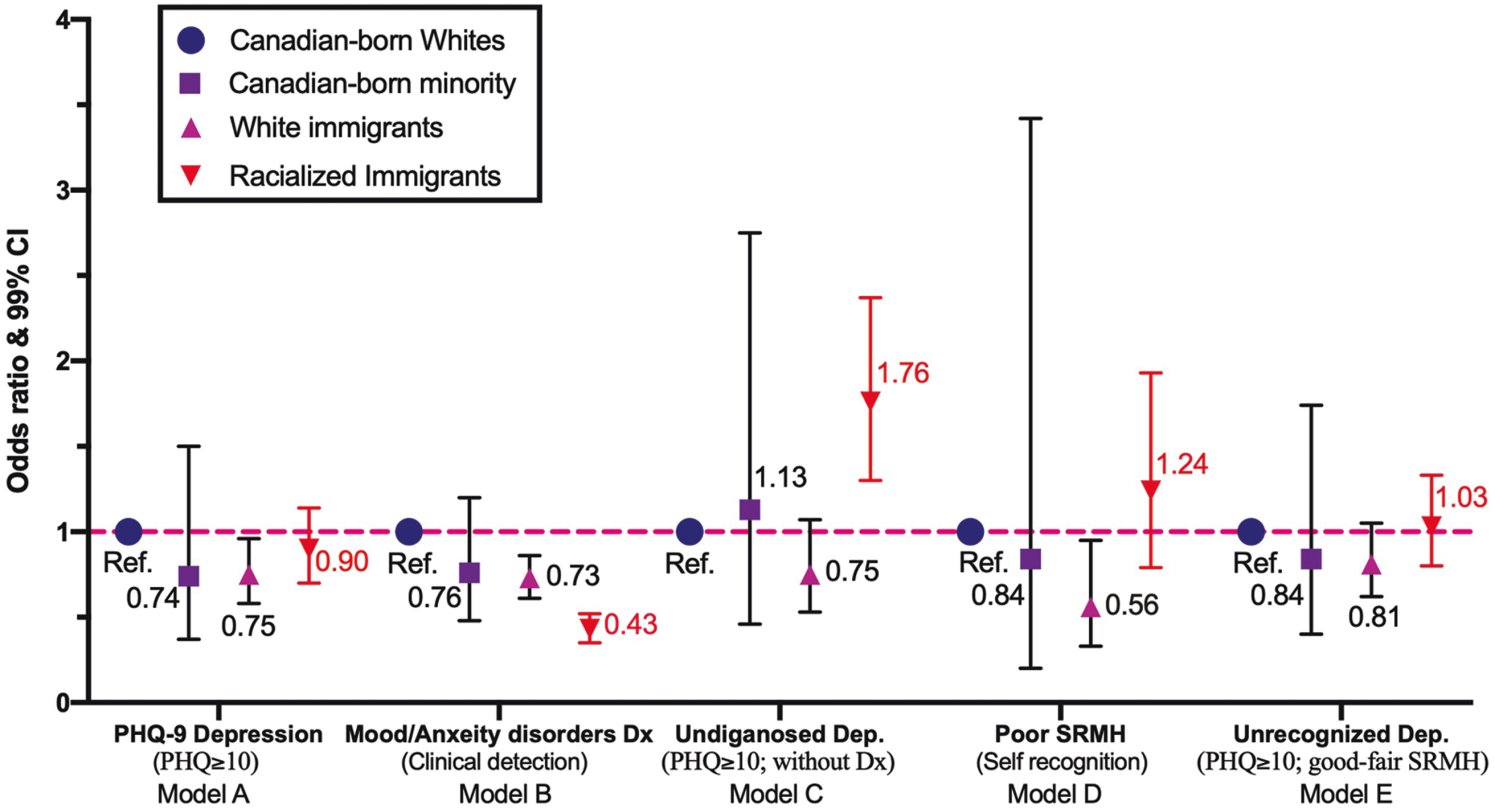
Disparities in depression symptoms (PHQ), mood/anxiety disorder diagnosis and undiagnosed depression in the Canadian Community Health Study 2015–2018, persons aged ≥45 (*N* = 28,951). *Notes*: Odds ratios were calculated based on multivariable logistic regression models. 99% CI = 99% confidence interval (*p* < .01 was considered statistically significant).

Figure 2 displays the results of logistic regression analyses to contrast symptom-rating and health professional-diagnosed measures (for full statistics, see Table 2). White immigrants stood out to be the only healthier group that was statistically significant, and they were 25% less likely to screen positive for the PHQ-9 depressive symptoms (Model A: adjusted odds ratio [AOR = 0.75, 99% Confidence interval [CI]: 0.58, 0.96) and 44% less likely to have poor SRMH (Model D: AOR = 0.56, 99% CI = 0.33, 0.95), compared to CB Whites. On the other hand, CB non-Whites and racialized immigrants did not statistically differ from CB Whites, and they shared a similar burden of PHQ-9 depressive symptoms and poor SRMH as CB Whites. Such nonsignificant results of the multivariate analysis in predicting PHQ-9 depression were in sharp contrast to the descriptive analysis, in which these three racialized and immigrant minority groups had lower rates of depression than CB Whites. This may suggest that the inclusion of several significant control variables (indicating other medically underserved groups) such as low-income households (a gradient pattern was observed in Table 2), persons relying on the emergency room as usual source of care and individuals with a weaker sense of community belonging may partly explain disparities in depressive symptoms among minority groups.

**Table 2.** Multivariable Logistic Regression in Predicting Mental Health Outcomes, CCHS 2015–2018, Persons Aged ≥45 (*N* = 28,951)

	Model A				Model B				Model C				Model D				Model E			
	Screen-positive depression				Mood/anxiety disorders Dx				Undiagnosed depression				Poor SRMH				Unrecognized depression			
	(PHQ-9 ≥ 10)				(Clinical detection)				(PHQ-9 ≥ 10; without Dx)				(Self-recognition)				(PHQ-9 ≥ 10; without poor SRMH)			
	Nagelkerke *R* = 0.216				Nagelkerke *R* = 0.165				Nagelkerke *R* = 0.118				Nagelkerke *R* = 0.233				Nagelkerke *R* = 0.176			
Explanatory variables	OR	99% CI		Sig.	OR	99% CI		Sig.	OR	99% CI		Sig.	OR	99% CI		Sig.	OR	99% CI		Sig.
Race–migration nexus (Ref. CB White)																				
** **CB non-White	0.74	0.37	1.50	.274	0.76	0.48	1.20	.119	1.13	0.46	2.75	.732	0.84	0.20	3.42	.742	0.84	0.40	1.74	.531
** **FB White	**0.75**	**0.58**	**0.96**	**.003**	**0.73**	**0.61**	**0.86**	**<.001**	0.75	0.53	1.07	.036	**0.56**	**0.33**	**0.95**	**.005**	0.81	0.62	1.05	.036
** **FB non-White	0.90	0.70	1.14	.244	**0.43**	**0.35**	**0.52**	**<.001**	**1.76**	**1.30**	**2.37**	**<.001**	1.24	0.79	1.93	.218	1.03	0.80	1.33	.735
Age (Ref. ≥75)																				
** **45–54	**1.54**	**1.14**	**2.07**	**<.001**	**2.20**	**1.77**	**2.74**	**<.001**	0.69	0.47	1.00	.010	0.64	0.39	1.06	.023	**1.47**	**1.07**	**2.02**	**.002**
** **55–64	**1.36**	**1.01**	**1.82**	**.007**	**1.94**	**1.57**	**2.41**	**<.001**	0.85	0.59	1.23	.258	0.66	0.40	1.07	.026	1.31	0.96	1.79	.025
** **65–74	0.94	0.69	1.28	.585	**1.59**	**1.28**	**1.98**	**<.001**	0.75	0.51	1.11	.059	**0.50**	**0.29**	**0.85**	**.001**	0.99	0.71	1.38	.948
Female (Ref. Male)	**1.55**	**1.34**	**1.81**	**<.001**	**1.72**	**1.54**	**1.91**	**<.001**	**1.57**	**1.27**	**1.93**	**<.001**	0.92	0.69	1.23	.460	**1.67**	**1.42**	**1.97**	**<.001**
Household income (Ref. ≥$80k)																				
** **<$20k	**2.91**	**2.24**	**3.79**	**<.001**	**2.98**	**2.44**	**3.63**	**<.001**	**1.52**	**1.04**	**2.23**	**.005**	**3.39**	**2.07**	**5.56**	**<.001**	**2.37**	**1.78**	**3.16**	**<.001**
** **$20k to <$40k	**1.71**	**1.36**	**2.16**	**<.001**	**1.64**	**1.39**	**1.94**	**<.001**	1.14	0.83	1.57	.300	**2.13**	**1.35**	**3.37**	**<.001**	**1.54**	**1.20**	**1.97**	**<.001**
** **$40k to <$60k	**1.55**	**1.24**	**1.93**	**<.001**	**1.56**	**1.34**	**1.82**	**<.001**	1.33	1.00	1.78	.011	**2.36**	**1.53**	**3.65**	**<.001**	**1.43**	**1.13**	**1.81**	**<.001**
** **$60k to <$80k	**1.42**	**1.14**	**1.78**	**<.001**	1.15	0.98	1.35	.024	1.09	0.80	1.48	.491	1.29	0.77	2.15	.202	**1.46**	**1.16**	**1.84**	**<.001**
Education (Ref. Postsecondary)																				
** **<Secondary school	1.20	0.98	1.47	.021	**0.85**	**0.73**	**0.99**	**.005**	**1.42**	**1.08**	**1.88**	**.001**	1.02	0.68	1.51	.913	**1.34**	**1.09**	**1.66**	**<.001**
** **Secondary school	**1.24**	**1.05**	**1.47**	**.001**	**0.84**	**0.74**	**0.95**	**<.001**	**1.33**	**1.06**	**1.68**	**.002**	**1.87**	**1.37**	**2.56**	**<.001**	**1.21**	**1.01**	**1.45**	**.007**
Rent home (Ref. Own home)	**1.31**	**1.10**	**1.56**	**<.001**	**1.21**	**1.06**	**1.38**	**<.001**	**1.36**	**1.06**	**1.74**	**.001**	1.31	0.95	1.80	.029	1.20	0.99	1.45	.015
Lack a regular care provider (Ref. No)	1.10	0.85	1.43	.324	**0.46**	**0.36**	**0.58**	**<.001**	**1.94**	**1.41**	**2.66**	**<.001**	1.01	0.63	1.64	.951	1.11	0.84	1.46	.350
Usual source of care (Ref. GP)																				
** **Hospital outpatient clinic	1.13	0.75	1.71	.452	1.06	0.79	1.43	.628	0.90	0.48	1.70	.673	1.02	0.45	2.31	.952	1.05	0.67	1.65	.763
** **Community health center	0.84	0.54	1.31	.314	0.98	0.74	1.30	.845	1.05	0.59	1.86	.832	0.55	0.20	1.53	.134	0.91	0.58	1.43	.582
** **Walk-in clinic	1.00	0.83	1.22	.957	1.03	0.90	1.18	.602	0.93	0.71	1.23	.525	1.03	0.72	1.48	.840	0.96	0.78	1.18	.618
** **Emergency room	**1.31**	**1.02**	**1.69**	**.006**	0.83	0.67	1.02	.020	**1.80**	**1.31**	**2.48**	**<.001**	0.88	0.52	1.47	.507	**1.38**	**1.06**	**1.81**	**.002**
** **Some other places	0.91	0.42	1.97	.745	0.92	0.54	1.57	.702	0.63	0.18	2.27	.356	1.45	0.46	4.58	.404	0.76	0.31	1.84	.420
** **Lack a USOC	0.96	0.72	1.27	.683	0.92	0.74	1.14	.327	1.03	0.70	1.51	.853	0.57	0.32	1.03	.014	0.98	0.72	1.34	.874
Languages at home (Ref. E/F)	**0.70**	**0.50**	**1.00**	**.009**	**0.77**	**0.59**	**1.00**	**.009**	0.96	0.64	1.43	.773	0.87	0.46	1.65	.578	0.77	0.54	1.09	.052
Community belonging (Ref. Strong)																				
** **Weak	**2.37**	**2.05**	**2.73**	**<.001**	**1.74**	**1.57**	**1.94**	**<.001**	**1.76**	**1.44**	**2.16**	**<.001**	**5.03**	**3.72**	**6.80**	**<.001**	**1.98**	**1.69**	**2.31**	**<.001**
** **Not stated	1.36	0.72	2.56	.210	0.91	0.55	1.50	.626	0.49	0.12	1.91	.174	**6.98**	**3.24**	**15.03**	**<.001**	0.47	0.17	1.31	.058
Relationship (Ref. Married)																				
** **Widowed	**1.20**	**1.00**	**1.44**	**.009**	**1.26**	**1.10**	**1.44**	**<.001**	0.85	0.65	1.11	.109	**1.77**	**1.26**	**2.48**	**<.001**	1.06	0.87	1.29	.483
** **Single	1.20	0.96	1.51	.037	**1.31**	**1.11**	**1.55**	**<.001**	1.08	0.78	1.49	.563	1.43	0.94	2.17	.028	1.19	0.94	1.52	.063
Perceived life stress (Ref. No)	**6.06**	**4.81**	**7.63**	**<.001**	**2.48**	**2.20**	**2.79**	**<.001**	**4.46**	**3.34**	**5.95**	**<.001**	**5.82**	**3.68**	**9.20**	**<.001**	**5.60**	**4.40**	**7.14**	**<.001**
BMI (Ref. Normal weight)																				
** **Underweight	1.41	0.79	2.51	.132	0.87	0.53	1.42	.454	1.73	0.82	3.67	.058	1.01	0.33	3.04	.989	1.56	0.85	2.87	.057
** **Overweight	**1.26**	**1.05**	**1.52**	**.001**	**1.34**	**1.18**	**1.52**	**<.001**	**1.30**	**1.01**	**1.68**	**.007**	0.99	0.69	1.40	.912	**1.31**	**1.08**	**1.59**	**<.001**
** **Obese—Class I, II, III	**1.91**	**1.59**	**2.29**	**<.001**	**1.74**	**1.53**	**1.99**	**<.001**	**1.82**	**1.41**	**2.35**	**<.001**	1.32	0.94	1.87	.036	**1.90**	**1.57**	**2.32**	**<.001**
** **Not stated	1.29	0.90	1.84	.071	0.92	0.70	1.23	.467	**1.87**	**1.19**	**2.96**	**<.001**	1.09	0.56	2.15	.731	1.33	0.91	1.96	.053
No past-week sports (Ref. Yes)	**1.88**	**1.61**	**2.20**	**<.001**	**1.23**	**1.11**	**1.37**	**<.001**	**1.68**	**1.36**	**2.09**	**<.001**	1.08	0.80	1.45	.506	**1.94**	**1.64**	**2.29**	**<.001**
Smoking (Ref. Nonsmoker)																				
** **Daily smoker	**1.79**	**1.51**	**2.13**	**<.001**	**1.77**	**1.56**	**2.02**	**<.001**	**1.40**	**1.09**	**1.81**	**.001**	**2.34**	**1.69**	**3.25**	**<.001**	**1.70**	**1.41**	**2.04**	**<.001**
** **Occasion smoker	1.20	0.83	1.74	.204	1.29	0.99	1.68	.012	1.12	0.65	1.92	.593	**2.59**	**1.49**	**4.49**	**<.001**	0.92	0.60	1.44	.645
Drinking (Ref. No)																				
** **Regular drinker	**0.81**	**0.68**	**0.96**	**.002**	**0.68**	**0.60**	**0.77**	**<.001**	1.23	0.96	1.59	.032	**0.55**	**0.40**	**0.77**	**<.001**	0.89	0.74	1.08	.124
** **Occasional drinker	0.89	0.72	1.09	.126	**0.82**	**0.70**	**0.95**	**<.001**	1.00	0.74	1.34	.965	0.85	0.60	1.23	.259	0.96	0.77	1.20	.660

*Notes*: *p* Value < .01 was considered statistically significant (bolded) and 99% confidence intervals (99% CI) were used to account for multiple testing.

BMI = Body mass index; CB = Canadian born; CCHS = Canadian Community Health Survey; Dx = diagnosis; E/F = English or French as primary language(s) at home; FB = foreign-born; GP = general practitioner; OR = odds ratio; PHQ-9 = Patient Health Questionnaire for major depression (PHQ-9 score ≥10 indicate moderate-to-severe symptoms); SRMH = self-rated mental health; USOC = usual source of care.

#### RQ2: Mood/anxiety disorders diagnosis

Racialized immigrants were 57% less likely to receive mood/anxiety disorders diagnosis (Model B: AOR = 0.43; 99% CI = 0.35, 0.52) than CB Whites. Similarly, White immigrants were 27% less likely to receive mood/anxiety disorders diagnosis (AOR = 0.73; 99% CI = 0.61, 0.86) than CB Whites. These associations were robust even after adjusting for many significant covariates, including socioeconomic factors and access to a regular care provider. In fact, those who lack a regular care provider were 54% less likely to receive a mood/anxiety disorders diagnosis (AOR = 0.46, 99% CI = 0.36, 0.58) than those who had one. For CB non-Whites, the odds of receiving mood/anxiety disorders diagnosis did not differ from CB White (*p* = .119). There are other significant control variables linked with increased odds of mood/anxiety disorders diagnosis including lower household income, weaker sense of community belonging, being widowed/single, having obesity, and daily smoking.

#### RQ3: Undiagnosed and unrecognized depressive symptoms

Racialized immigrants were the only population with 76% greater odds of having undiagnosed depression than CB Whites, even after controlling for all covariates (Model C: odds ratio [AOR] = 1.76, 99% CI: 1.30–2.37). There were no significant associations with undiagnosed depression among CB non-Whites and White immigrants. For unrecognized depression (Model E), there were no significant racial–migration disparities in the fully adjusted model. In addition, there were certain modifiable risk factors (all *p* < .001) identified for undiagnosed depression (see Table 2, Model C), such as lack of a regular care provider (AOR = 1.94, 99% CI: 1.41–2.66), emergency room as the usual source of care (AOR = 1.80, 99% CI: 1.31–2.48), weaker sense of community belonging (AOR = 1.76, 99% CI: 1.44–2.16), perceived stressful life (AOR = 4.46, 99% CI: 3.34–5.95), and having obesity (AOR = 1.82, 99% CI: 1.41–2.35).

#### Sensitivity tests: Stratified analyses by clinical detection

**Figure 3.**
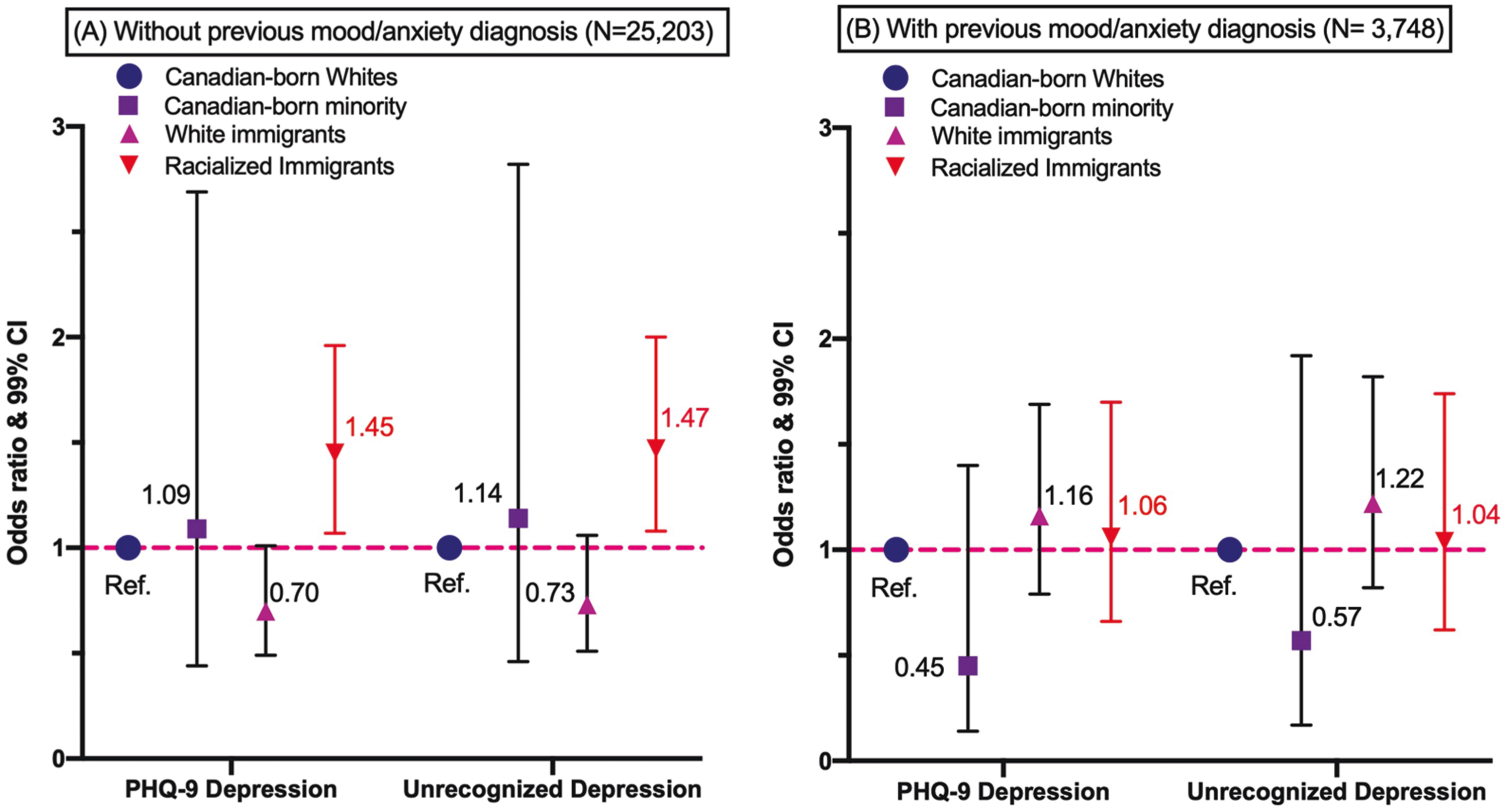
Stratified analysis: Disparities in depression symptoms (PHQ-9) and unrecognized depression stratified by the presence/absence of mood/anxiety disorder diagnosis (M/A-Dx). *Notes*: Odds ratios were calculated based on multivariable logistic regression models. 99% CI = 99% Confidence Interval (*p* < .01 was considered statistically significant).

More nuances were found when repeating the logistic regression analysis via stratifying the sample by the clinical detection of M/A-Dx (see Figure 3; for full statistics see Supplementary Material 1). Among the subpopulation without a previous M/A-Dx (*N* = 25,203), racialized immigrants stood out to be the only mentally disadvantaged group who were more likely to screen positive for PHQ-9 depression (AOR = 1.45, 99% CI: 1.15–1.82) and to have unrecognized depression (AOR = 1.47, 99% CI: 1.08–2.00) than CB Whites. Canadian-born non-Whites and FB Whites did not differ from CB Whites for PHQ depression and unrecognized depression. Among the subpopulation with a previous M/A-Dx (*N* = 3,748), there were no significant racial–migration disparities in these two outcome measures (*p* > .05) after full adjustment. The differences between subpopulations confirmed that the stratifier (i.e., prior clinical detection) was a confounding factor for racialized immigrants to recognize mental health problems, possibly due to heightened mental health literacy among those who received diagnostic care.

## Discussion

Overall, the current investigation reveals that an estimated 5.6% of Canadians aged 45 years and older were screened positive for PHQ-9 depressive symptoms, among whom 43% had not obtained a mood/anxiety disorders diagnosis in their lifetime, suggesting that late-life depression is at risk of being underestimated in the general aging population. This is consistent with a previous epidemiological study, which found that almost half of Canadians with symptoms compatible with a mood disorder reported no previous diagnosis (Pelletier et al., 2017). Through examining symptomatic individuals by diagnosis status, our study implies that measures of mental disorder diagnoses from health professionals in clinical settings may underestimate the actual population burden of depression in the community (Fan et al., 2009). As such, this study calls for a paradigm shift—for medically underserved populations (e.g., those who lack a regular care provider and those who rely on emergency room as a usual source of care)—measures of professional-diagnosed mental disorders should be treated as proxies for receiving diagnostic care services (Akincigil et al., 2012), instead of preciseestimation for mental health prevalence in the community. In the Canadian context, even though the diagnostic process for mental health conditions is heavily dependent on publicly funded physician-provided services, the under-detection of depression was more pronounced among racialized immigrants but not among White immigrants in mid-to-late life.

Under-detection of depression in the community may reflect a joint provider–client problem (Epstein et al., 2010; Garrard et al., 1998), as the depression diagnostic pathway is composed of multiple settings and stages. Fortunately, no racial–migration inequities in access to a regular care provider were found in the bivariate analysis (*p* = .15), primarily due to the Canadian universal health coverage. As such, even though access to the Canadian health care system appears not to be a major problem for diverse racial–migration groups, two possible scenarios potentially explaining under-detection may be: (a) on the patient side: older adults with depressive symptoms did not see a regular care provider for mental health concerns; or (b) on the provider side: older adults with depressive symptoms did see care providers, but the symptomology associated with depression was not detected by health professionals or such symptoms did not reach the clinical threshold of depression (e.g., experiencing significant dysfunction in daily life).

For the client/patient side, barriers to mental health care-seeking could still range from attitudinal obstacles (Lin, 2022a), such as mental health stigma (Conner et al., 2010), racial variations in mental health literacy (Na et al., 2016), self-awareness of depressive symptoms (Kim et al., 2011), perceived need for mental health care (Villatoro et al., 2018), mistrust between health care professionals and patients (Atdjian & Vega, 2005; Farid et al., 2020), to more structural impediments including lack of culturally responsive services (Sentell et al., 2007), and discrimination (Edge & Newbold, 2013). For instance, research has found depressed older adults endorsed a high level of internalized stigma and they were less likely to seek mental health treatment, particularly those from racialized communities (Conner et al., 2010). Among individuals without previous clinical detection in this study, racialized immigrants were less likely than CB Whites to recognize their current moderate-to-severe depressive symptoms as signs of poor mental health. It may be that immigrants with certain cultural beliefs have distinct explanatory models of depression, in which emotional discomfort was seen as a normal part of life rather than an episode of mental illness (Kleinman, 2004) or it was considered as a socio-moral issue that should be consulted with family members, community leaders, spiritual or other traditional healers (Kirmayer, 2001; Kirmayer et al., 2011).

For the provider/clinician side, the lower prevalence estimates for mental disorder diagnosis rates among racialized immigrants may reflect inadequate detection of psychiatric conditions by primary care practitioners (Borowsky et al., 2000) and under-referral to psychiatric care in clinical settings (Atdjian & Vega, 2005). Cross-cultural clinical encounters are at greater risk of misinterpretation of psychological symptoms (Chen et al., 2009; Kirmayer, 2001). Prior research has found ethnic, cultural, and nativity disparities in clinical presentation and symptom expression (e.g., somatization) of depression (Dreher et al., 2017; Lanzara et al., 2018), such as the tendency to articulate somatic (bodily) instead of affective complaints among racialized immigrant clients (Li & Browne, 2000; Chiriboga et al., 2005). Prior U.S. research has found race/ethnicity influenced physician recognition of depression in a way that racialized patients had physicians who were less oriented toward providing counseling for depression than physicians of White patients (Borowsky et al., 2000). As such, this study highlights the need for mental health practitioners to develop race/ethnicity-specific and culturally responsive strategies in assessing depression for diverse aging populations, such as validating patients’ lived experiences, untangling culturally patterned ways (e.g., idioms) of expressing emotional difficulties (Kirmayer, 2001), translating culturally coded symptoms (Kleinman, 2004) reducing stigma and decoding wider social meanings of distress (Epstein et al., 2010).

Our study offers some insights into immigrant health research. It is the first attempt, to the best of our knowledge, to explore multiple mental health constructs: racialized immigrants were less likely than CB Whites to receive a health professional’s diagnosis of mood disorders or anxiety disorders, in spite of the equivalent prevalence of current depressive symptoms; and consequently, they were more likely to have undiagnosed depression (Wang et al., 2019). These findings provide a counterdiscourse that challenges previous research relying solely on diagnoses in estimating the burden of mental disorders (Aglipay et al., 2013; Chiu et al., 2018; Nwoke et al., 2020). Moreover, while racialized immigrants did not differ from CB Whites in experiencing poor SRMH and PHQ-9 depression, White immigrants were significantly less likely than CB Whites to report poor SRMH and PHQ-9 depression. These contrasting pattern implies that the HIE for mental health occurs mainly among White immigrants but not racialized immigrants in the Canadian aging population, suggesting that such mental health advantage is not a monolithic phenomenon but is contingent upon race/ethnicity (Brown, 2018; Vang et al., 2017). Notably, distinct from essentialist acculturation-related research the present study found that those traditionally deemed to be acculturation indicators—primary languages spoken at home (e.g., Sentell et al., 2007) and sense of community belonging (e.g., Berry & Hou, 2021)—did not attenuate racialized immigrants’ susceptibility in undiagnosed depression, suggesting that race–migration nexus may reflect other underlying experiences of psychosocial stressors (e.g., structural inequities, racial discrimination) than cultural factors (Veenstra, 2009). Thus, besides cultural sensitivity, structural competence training is also needed for mental health professionals to look beyond clinical interactions by considering macro-level explanations for mental health concerns among immigrants (How et al., 2021).

## Limitations

By using population-based data, this study has several strengths, including its large sample size, its ability to include the intersecting measure of race and migration, and its attention to the cross-referencing evaluation of diverse mental health indicators in the aging populations. However, the study has several methodological limitations. First, the causal relationship between depression and its risk factors cannot be inferred as the data were cross-sectional. Second, due to the confidentiality protection in the PUMF data, many important measurements that may disentangle the mechanisms, dynamics, and care-seeking processes could not be examined, such as migration admission class, racial composition, and measures of discrimination. Third, the current pooled sample did not account for the time effect. To provide more precise estimations, it is recommended that weights should be further rescaled by a constant factor to cover the combined periods of individual cycles (Thomas & Wannell, 2009). Because the CCHS adopts a complex sample design, a bootstrap variance estimation would have been preferable. However, given the CCHS public-use data set did not provide bootstrap weights, applying normalized weights in the current study, as a stop-gap approach, may underestimate the true variance of the estimates (Statistics Canada, 2014).

It should be noted that immigrants (aged ≥45 years) in the present sample were predominantly established immigrants (94%, ≥10 years, unweighted *n* = 3,781) with only 6% being recent immigrants (unweighted *n* = 246) who arrived in less than 10 years preceding the CCHS survey. Therefore, the immigrant cohort effect (i.e., years since immigration) could not be concurrently tested as the inclusion of such a variable will lead to model redundancies. Because the CCHS data only specify landed immigrant status (i.e., permanent residents), those with precarious migration status—that is, foreign-born temporary residents (on work or study visas) as well as undocumented immigrants—could not be examined in the current study and they are more susceptible to mental health concerns due to restrictive entry policies (Juárez et al., 2019).

In terms of measurement bias, the PHQ-9 was not a substitute for a thorough clinical evaluation by a trained health care professional and was not equivalent to a structured interview-based diagnosis of depression, despite its 88% sensitivity of major depression; thus, the PHQ-positive symptom scores ≥10 may be at risk of false positive scenario (over-detection or misidentifications), in which patients who exceed the cut-off score may not necessarily be clinically depressed or need a diagnosis. In addition, the self-report diagnoses of mental disorders alone may under-estimate clinical detection rates (O’Donnell et al., 2016), and a comprehensive measure would be preferable to encapsulate mental health care utilizations such as visits to mental health specialists, use of psychotherapy, and antidepressant medications (Garrard et al., 1999); yet these indicators were not consistently captured in the CCHS data. For racialized immigrants with diverse cultures, questions also remain whether accessing alternative ways of healing (e.g., herbalists) for mental health reasons should be counted as clinical detection in a Western healthcare system (Lin, 2023). As such, the measure of undiagnosed depression may just serve as a rough community-level estimation. Future studies could investigate racial-nativity disparities in mental health by comparing survey and administrative data sources and by using a longitudinal design.

## Conclusions

Late-life depression is a serious but under-recognized health issue. This study untangles the joint impact of race and migration as markers of mental health inequalities in symptom prevalence, diagnosis rates, and undiagnosed depression in a population-based sample of older Canadians. The burden of undiagnosed depression disproportionately affects older racialized immigrants, despite a publicly funded health system for mental health diagnosis in Canada. This result underscores the necessity to move beyond a simplistic focus on health professionals’ diagnoses in examining mental health prevalence. The “healthy immigrant effect” on late life mental health may be mainly driven by the healthier profile of White immigrants, and partly attributable to underreporting of professional-diagnosed mental health conditions among racialized immigrants, who may experience psychosocial barriers to timely mental health assessment in Canada. It appears that under-detection by health providers and possible under-recognition of mental health problems by immigrants themselves may account for the incorrect conclusion of a mental health advantage for racialized immigrants. In addition, undiagnosed depression was associated with health care inaccessibility (i.e., lack of a regular doctor, emergency room as usual source of care), which could serve as important points of prevention and intervention to address mental health inequalities in a multiracial aging society.

## Supplementary Material

gbad104_suppl_Supplementary_Material

## Data Availability

The public-use microdata file of the Canadian Community Health Survey is available to Canadian researchers via Statistics Canada’s Data Liberation Initiative and to international researchers by request at dli-idd@statcan.gc.ca from Statistics Canada. The public-use data are completely de-identified and publicly available with necessary suppression methods to protect confidentiality.
