# Supplementary material for: Healthy Immigrant Effect or Under-Detection? Examining Undiagnosed and Unrecognized Late-Life Depression for Racialized Immigrants and Nonimmigrants in Canada
Source: J Gerontol B Psychol Sci Soc Sci. 2023 Jul 27;79(3):gbad104. doi: 10.1093/geronb/gbad104 (PMC11036341; doi:10.1093/geronb/gbad104)
Supplement: gbad104_suppl_Supplementary_Material [file gbad104_suppl_supplementary_material.docx]

**Supplemental Material**

**Stratified Analyses: Disparities in Depression Symptoms (PHQ-9) and Unrecognized Depression Stratified by the Presence/Absence of Mood/Anxiety Disorder diagnosis (Clinical detection) – Full statistics for Figure 3**

|  | **Without previous mood/anxiety diagnosis (Dx) N=25,203** | | | | | | | | | | |  | | **With previous mood/anxiety diagnosis (Dx) N=3748** | | | | | | | | | | | |  |
| --- | --- | --- | --- | --- | --- | --- | --- | --- | --- | --- | --- | --- | --- | --- | --- | --- | --- | --- | --- | --- | --- | --- | --- | --- | --- | --- |
|  | **Screen-positive Depression** | | | |  | | **Unrecognized Depression** | | | | |  | | **Screen-positive Depression** | | | | |  | | **Unrecognized Depression** | | | | |  |
|  | **(PHQ≥10)** | | | |  | | **(PHQ≥10; Good SRMH)** | | | | |  | | **(PHQ≥10)** | | | | |  | | **(PHQ≥10; Good SRMH)** | | | | |  |
|  | Nagelkerke R=0.149 | | | |  | | Nagelkerke R=0.15 | | | | |  | | Nagelkerke R=0.24 | | | | |  | | Nagelkerke R=0.152 | | | | |  |
|  | OR | 99%CI | | Sig. | |  | | OR | 99%CI | | Sig. | |  | | OR | 99%CI | | Sig. | |  | | OR | 99%CI | | Sig. | |
| **Race-migration nexus (Ref. CB White)** |  |  |  |  | |  | |  |  |  |  | |  | |  |  |  |  | |  | |  |  |  |  | |
| CB non-White | 1.09 | 0.44 | 2.69 | 0.813 | |  | | 1.14 | 0.46 | 2.82 | 0.713 | |  | | 0.45 | 0.14 | 1.40 | 0.069 | |  | | 0.57 | 0.17 | 1.92 | 0.230 | |
| FB White | 0.70 | 0.49 | 1.01 | 0.011 | |  | | 0.73 | 0.51 | 1.06 | 0.028 | |  | | 1.16 | 0.79 | 1.69 | 0.323 | |  | | 1.22 | 0.82 | 1.82 | 0.199 | |
| FB non-White | **1.45** | **1.07** | **1.96** | **0.002** | |  | | **1.47** | **1.08** | **2.00** | **0.001** | |  | | 1.06 | 0.66 | 1.70 | 0.769 | |  | | 1.04 | 0.62 | 1.74 | 0.848 | |
| **Age (Ref. ≥75)** |  |  |  |  | |  | |  |  |  |  | |  | |  |  |  |  | |  | |  |  |  |  | |
| 45-54 | 0.84 | 0.57 | 1.24 | 0.251 | |  | | 0.91 | 0.61 | 1.36 | 0.524 | |  | | 1.61 | 0.97 | 2.67 | 0.015 | |  | | 1.66 | 0.96 | 2.88 | 0.017 | |
| 55-64 | 1.02 | 0.70 | 1.48 | 0.907 | |  | | 1.10 | 0.75 | 1.62 | 0.528 | |  | | 1.10 | 0.66 | 1.82 | 0.632 | |  | | 1.08 | 0.63 | 1.88 | 0.705 | |
| 65-74 | 0.85 | 0.57 | 1.25 | 0.270 | |  | | 0.88 | 0.59 | 1.33 | 0.438 | |  | | 0.69 | 0.40 | 1.18 | 0.074 | |  | | 0.86 | 0.48 | 1.54 | 0.504 | |
| **Female (Ref. Male)** | **1.71** | **1.38** | **2.11** | **<0.001** | |  | | **1.76** | **1.42** | **2.19** | **<0.001** | |  | | 0.99 | 0.78 | 1.25 | 0.881 | |  | | 1.12 | 0.87 | 1.45 | 0.251 | |
| **Household income (Ref. ≥$80k)** |  |  |  |  | |  | |  |  |  |  | |  | |  |  |  |  | |  | |  |  |  |  | |
| < $20k | **2.10** | **1.42** | **3.09** | **<0.001** | |  | | **2.00** | **1.34** | **2.97** | **<0.001** | |  | | **2.04** | **1.38** | **3.02** | **<0.001** | |  | | **1.70** | **1.11** | **2.60** | **0.001** | |
| $20k to <$40k | 1.24 | 0.90 | 1.72 | 0.089 | |  | | 1.18 | 0.85 | 1.65 | 0.198 | |  | | **1.66** | **1.17** | **2.36** | **<0.001** | |  | | **1.60** | **1.10** | **2.34** | **0.001** | |
| $40k to <$60k | 1.44 | 1.07 | 1.93 | 0.002 | |  | | **1.44** | **1.07** | **1.94** | **0.002** | |  | | 1.32 | 0.93 | 1.87 | 0.044 | |  | | 1.13 | 0.77 | 1.67 | 0.400 | |
| $60k to <$80k | 1.08 | 0.79 | 1.47 | 0.533 | |  | | 1.05 | 0.76 | 1.44 | 0.719 | |  | | **2.07** | **1.45** | **2.96** | **<0.001** | |  | | **2.34** | **1.62** | **3.38** | **<0.001** | |
| **Education (Ref. Post-secondary)** |  |  |  |  | |  | |  |  |  |  | |  | |  |  |  |  | |  | |  |  |  |  | |
| <Secondary school | **1.38** | **1.04** | **1.83** | **0.004** | |  | | **1.38** | **1.04** | **1.85** | **0.004** | |  | | 1.20 | 0.88 | 1.62 | 0.131 | |  | | 1.37 | 1.00 | 1.89 | 0.011 | |
| Secondary school | **1.32** | **1.04** | **1.67** | **0.003** | |  | | **1.31** | **1.03** | **1.67** | **0.004** | |  | | **1.41** | **1.09** | **1.83** | **0.001** | |  | | 1.20 | 0.90 | 1.59 | 0.104 | |
| **Rent home (Ref. own home)** | **1.47** | **1.15** | **1.89** | **<0.001** | |  | | **1.45** | **1.12** | **1.86** | **<0.001** | |  | | 1.02 | 0.78 | 1.35 | 0.822 | |  | | 0.88 | 0.65 | 1.19 | 0.270 | |
| **Lack of a regular care provider (Ref. No)** | **1.60** | **1.15** | **2.22** | **<0.001** | |  | | **1.55** | **1.11** | **2.18** | **0.001** | |  | | 1.32 | 0.81 | 2.14 | 0.144 | |  | | 0.84 | 0.48 | 1.47 | 0.419 | |
| **Usual Source of Care (Ref. GP)** |  |  |  |  | |  | |  |  |  |  | |  | |  |  |  |  | |  | |  |  |  |  | |
| Hospital outpatient clinic | 0.93 | 0.49 | 1.77 | 0.771 | |  | | 0.79 | 0.39 | 1.59 | 0.380 | |  | | 1.23 | 0.68 | 2.23 | 0.370 | |  | | 1.36 | 0.73 | 2.54 | 0.201 | |
| Community health centre | 1.03 | 0.57 | 1.84 | 0.905 | |  | | 1.07 | 0.60 | 1.91 | 0.773 | |  | | 0.73 | 0.37 | 1.45 | 0.243 | |  | | 0.78 | 0.38 | 1.61 | 0.374 | |
| Walk-in clinic | 0.97 | 0.73 | 1.29 | 0.798 | |  | | 0.91 | 0.68 | 1.21 | 0.379 | |  | | 1.03 | 0.76 | 1.38 | 0.824 | |  | | 1.06 | 0.77 | 1.45 | 0.655 | |
| Emergency room | **1.74** | **1.25** | **2.43** | **<0.001** | |  | | **1.77** | **1.26** | **2.47** | **<0.001** | |  | | 1.16 | 0.76 | 1.78 | 0.368 | |  | | 1.09 | 0.69 | 1.72 | 0.612 | |
| Some other place | 0.69 | 0.19 | 2.52 | 0.462 | |  | | 0.73 | 0.20 | 2.67 | 0.530 | |  | | 1.19 | 0.40 | 3.55 | 0.677 | |  | | 0.76 | 0.22 | 2.65 | 0.574 | |
| Lack a USOC | 0.99 | 0.67 | 1.46 | 0.941 | |  | | 1.05 | 0.71 | 1.55 | 0.766 | |  | | 1.06 | 0.67 | 1.67 | 0.764 | |  | | 0.91 | 0.55 | 1.52 | 0.638 | |
| **Languages at home (Ref. E/F)** | 0.94 | 0.63 | 1.41 | 0.703 | |  | | 0.98 | 0.65 | 1.47 | 0.878 | |  | | 0.41 | 0.19 | 0.88 | 0.003 | |  | | 0.53 | 0.24 | 1.17 | 0.038 | |
| **Belongingness (Ref. strong)** |  |  |  |  | |  | |  |  |  |  | |  | |  |  |  |  | |  | |  |  |  |  | |
| Weak | **1.98** | **1.61** | **2.44** | **<0.001** | |  | | **1.93** | **1.57** | **2.38** | **<0.001** | |  | | **2.27** | **1.82** | **2.83** | **<0.001** | |  | | **1.56** | **1.23** | **1.98** | **<0.001** | |
| Not stated | 0.50 | 0.13 | 1.97 | 0.192 | |  | | 0.52 | 0.13 | 2.05 | 0.216 | |  | | **3.11** | **1.23** | **7.83** | **0.002** | |  | | 0.38 | 0.09 | 1.73 | 0.102 | |
| **Relationship (Ref. Married)** |  |  |  |  | |  | |  |  |  |  | |  | |  |  |  |  | |  | |  |  |  |  | |
| Widowed | 0.92 | 0.70 | 1.20 | 0.401 | |  | | 0.88 | 0.67 | 1.17 | 0.250 | |  | | 1.31 | 1.00 | 1.72 | 0.011 | |  | | 1.05 | 0.78 | 1.41 | 0.654 | |
| Single | 1.09 | 0.78 | 1.52 | 0.515 | |  | | 1.12 | 0.80 | 1.57 | 0.390 | |  | | 1.00 | 0.71 | 1.41 | 0.988 | |  | | 0.99 | 0.68 | 1.43 | 0.930 | |
| **Perceived life stress (Ref. No)** | **5.05** | **3.77** | **6.75** | **<0.001** | |  | | **5.14** | **3.81** | **6.94** | **<0.001** | |  | | **4.80** | **3.27** | **7.04** | **<0.001** | |  | | **3.78** | **2.50** | **5.73** | **<0.001** | |
| **BMI (Ref. Normal weight)** |  |  |  |  | |  | |  |  |  |  | |  | |  |  |  |  | |  | |  |  |  |  | |
| Underweight | 1.63 | 0.75 | 3.52 | 0.103 | |  | | 1.77 | 0.82 | 3.83 | 0.057 | |  | | 1.16 | 0.44 | 3.06 | 0.685 | |  | | 1.29 | 0.47 | 3.57 | 0.519 | |
| Overweight | **1.39** | **1.08** | **1.80** | **0.001** | |  | | **1.42** | **1.09** | **1.85** | **0.001** | |  | | 0.92 | 0.69 | 1.22 | 0.429 | |  | | 0.96 | 0.71 | 1.31 | 0.748 | |
| Obese - Class I, II, III | **2.07** | **1.60** | **2.70** | **<0.001** | |  | | **2.15** | **1.64** | **2.81** | **<0.001** | |  | | 1.33 | 1.00 | 1.75 | 0.009 | |  | | 1.24 | 0.92 | 1.68 | 0.060 | |
| Not stated | **1.85** | **1.16** | **2.96** | **0.001** | |  | | **1.99** | **1.24** | **3.18** | **<0.001** | |  | | 0.92 | 0.51 | 1.66 | 0.710 | |  | | 0.81 | 0.41 | 1.57 | 0.404 | |
| **No past-week sports (Ref. Yes)** | **1.74** | **1.40** | **2.16** | **<0.001** | |  | | **1.85** | **1.48** | **2.31** | **<0.001** | |  | | **1.88** | **1.48** | **2.39** | **<0.001** | |  | | **1.87** | **1.44** | **2.42** | **<0.001** | |
| **Smoking (Ref. Non-smoker)** |  |  |  |  | |  | |  |  |  |  | |  | |  |  |  |  | |  | |  |  |  |  | |
| Daily smoker | **1.65** | **1.27** | **2.15** | **<0.001** | |  | | **1.67** | **1.28** | **2.18** | **<0.001** | |  | | **1.37** | **1.07** | **1.76** | **0.001** | |  | | 1.27 | 0.97 | 1.66 | 0.021 | |
| Occasion smoker | 1.19 | 0.69 | 2.07 | 0.412 | |  | | 1.24 | 0.71 | 2.15 | 0.329 | |  | | 0.81 | 0.47 | 1.41 | 0.321 | |  | | **0.47** | **0.23** | **0.96** | **0.007** | |
| **Drinking (Ref. No)** |  |  |  |  | |  | |  |  |  |  | |  | |  |  |  |  | |  | |  |  |  |  | |
| Regular drinker | 1.10 | 0.85 | 1.42 | 0.351 | |  | | 1.13 | 0.87 | 1.47 | 0.227 | |  | | **0.76** | **0.58** | **1.00** | **0.009** | |  | | 0.80 | 0.60 | 1.07 | 0.050 | |
| Occasional drinker | 0.97 | 0.72 | 1.32 | 0.806 | |  | | 0.95 | 0.69 | 1.30 | 0.674 | |  | | 0.90 | 0.66 | 1.22 | 0.368 | |  | | 1.03 | 0.74 | 1.43 | 0.809 | |

**Notes:** P-value <0.01 was considered statistically significant (bolded) and 99% confidence intervals (99% CI) were used to account for multiple testing. PHQ-9=Patient Health Questionnaire for major depression (PHQ-9 score≥10 indicate moderate-to-severe symptoms). CCHS= Canadian Community Health Survey. CB=Canadian-born. FB=Foreign-born. GP=General practitioner. SRMH=self-rated mental health. E/F=English or Frech as primary language(s) at home. USOC=Usual Source of Care. BMI=Body mass index.
